# Roles and effectiveness of lay community health workers in the prevention of mental, neurological and substance use disorders in low and middle income countries: a systematic review

**DOI:** 10.1186/1472-6963-13-412

**Published:** 2013-10-13

**Authors:** Byamah Brian Mutamba, Nadja van Ginneken, Lucy Smith Paintain, Simon Wandiembe, David Schellenberg

**Affiliations:** 1 Butabika National Mental Referral Hospital, P.O. Box 7017, Kampala, Uganda; 2 Department of Population Health, London School of Hygiene and Tropical Medicine, Keppel Street, London WC1E 7HT, England; 3 Department of Disease Control, London School of Hygiene and Tropical Medicine, Keppel Street, London WC1E 7HT, England; 4 Department of Medical Statistics, London School of Hygiene and Tropical Medicine, Keppel Street, London WC1E 7HT, England; 5 Department of Disease Control, Faculty of Infectious and Tropical diseases, London School of Hygiene and Tropical Medicine, Keppel Street, London, WC1E 7HT, England

**Keywords:** Prevention, Lay community health workers, Mental, Neurological and substance use disorders, Developing countries, Systematic review

## Abstract

**Background:**

It has been suggested that lay community health workers (LHWs) could play a role in primary and secondary prevention of Mental, Neurological and Substance use (MNS) disorders in low resourced settings. We conducted a systematic review of the literature with the aim of assessing the existing evidence base for the roles and effectiveness of LHWs in primary and secondary prevention of MNS disorders in low and middle income countries (LMICs).

**Methods:**

Internet searches of relevant electronic databases for articles published in English were done in August 2011 and repeated in June 2013. Abstracts and full text articles were screened according to predefined criteria. Authors were asked for additional information where necessary.

**Results:**

A total of 15 studies, 11 of which were randomised, met our inclusion criteria. Studies were heterogeneous with respect to interventions, outcomes and LHWs’ roles. Reduction in symptoms of depression and improved child mental development were the common outcomes assessed. Primary prevention and secondary prevention strategies were carried out in 11 studies and 4 studies respectively .There was evidence of effectiveness of interventions however, most studies (n = 13) involved small sample sizes and all were judged to have an unclear or high risk of bias.

**Conclusions:**

LHWs have the potential to provide psychosocial and psychological interventions as part of primary and secondary prevention of MNS disorders in LMICs, but there is currently insufficient robust evidence of effectiveness of LHW led preventive strategies in this setting. More studies need to be carried out in a wider range of settings in LMICs that control for risk of bias as far as possible, and that also collect indicators relating to the fidelity and cost of interventions.

## Background

Community based health care is becoming one of the preferred approaches to increasing accessibility to public health services, especially in low and middle income countries (LMICs) [1]. It commonly involves use of community health workers (CHWs) who in most cases are lay people in communities trained to provide some part of the health services. Lay community health workers (LHWs) are used to fill the gap left by lack of sufficient human resources in the public sector, to link communities to the formal health service through ‘task shifting’ [2,3]. Task shifting, increasingly referred to as ‘task sharing’, is defined as ‘delegating tasks to existing or new cadres with either less training or narrowly tailored training for the required service’ [4]. It is primarily about the rational redistribution of tasks among existing health workforce teams in order to make the most efficient use of the health workers in the system [1].

The renewed focus on the use of LHWs has its rationale primarily in the recognition that service needs, particularly in remote and underprivileged communities, are not met by existing health services [2]. These LHWs may receive training, which is recognized by the health services and national certification authority, but this training does not form part of a tertiary education certificate [2,3]. Those who undertake specific training to perform clearly delineated tasks, can be deployed much faster than the more highly trained cadres and can play an important role in complementing and supporting the services provided by other health workers [1]. This approach has been widely used in maternal and child health, and communicable disease control programmes particularly for malaria, HIV and tuberculosis [1,3].

There is an increased demand for a similar approach to be used in mental health service provision including prevention programmes [5], particularly in LMICs where there are large shortages of mental health professionals relative to the burden of Mental, Neurological and Substance use (MNS) disorders [6]. LMICs account for about 85% of the world’s population, and almost three quarters of the global burden of neuropsychiatric disorders. Despite the significant burden of MNS disorders, the treatment gaps for persons with MNS disorders are highest in these countries with treatment rates ranging from 35% to 50% of those diagnosed [6,7].

Mental disorder prevention aims at “reducing incidence, prevalence, recurrence of mental disorders, the time spent with symptoms, or the risk condition for a mental illness, preventing or delaying recurrences and also decreasing the impact of illness in the affected person, their families and the society” [8].

Prevention can be classified into primary, secondary and tertiary [8]. Primary prevention targets both those populations who may be at risk (selective and indicated) and those who are not at risk (universal) for MNS disorders [8-10]. Secondary prevention includes early detection, treatment and referral of cases with the aim of arresting the disorder before it fully develops [9-11]. Tertiary prevention interventions aim at reducing disability, enhancing rehabilitation and preventing relapses and recurrences of the illness (8, 10). In this review, primary prevention was defined as programmes that had services directed toward reducing incidence or prevalence of MNS disorders, and secondary prevention as programmes involved in the early identification, referral and treatment of persons with symptoms of a MNS disorder aimed at arresting a disorder before it fully develops [8,12]. Tertiary prevention was not studied as it had been included in a previous review [13]. These definitions were chosen to fit in with suggested LHW roles [5].

It has been suggested that LHWs could play a role in primary and secondary prevention of MNS disorders through health education, case identification and referral [5]. Previous systematic reviews have explored the effectiveness of interventions led by LHWs on health care delivery, improvement in health outcomes and reduction in mortality [3,14]. None are known to have examined the evidence for the effectiveness of using LHWs in MNS disorder prevention strategies in LMICs.

This review complements an existing Cochrane review which examines the effectiveness of non-specialist health workers in delivering mental health care in LMICs and includes tertiary but not primary or secondary prevention strategies [13]. The objectives of this systematic review were to identify and describe the roles, and assess the effectiveness of using LHWs in the primary and secondary prevention of MNS disorders in LMICs, with the intention that information generated will inform future community mental health care initiatives in similar settings.

## Methods

### Search strategy for identification of studies

Search terms were developed by dividing the research question into four concepts; a) lay community health workers b) mental, neurological and substance use disorders, c) prevention strategies and d) low and middle income countries. Key words and subject headings were identified from the literature on the subject. For each concept, key words with truncation or wild card symbols and medical subject headings (MeSH) were combined with “OR”. The four concepts were then combined with “AND” to generate the final list of records identified.

The search strategy was initially developed and run in MEDLINE, then adapted as required for the following complementary electronic databases: EMBASE, PsycINFO, Global Health, Cochrane Library and Cochrane Register of Controlled trials, ELDIS, Africa Wide Information, IMEMR, IMSEAR, LILACS, MedCarib and WPRIM. The above databases were chosen to ensure a comprehensive literature search relevant to the study question. No restriction was placed on date of publication. Searches were initially conducted in August 2011 and were then repeated in June 2013.

### Inclusion and exclusion criteria

#### Types of participants -clients

The systematic review’s focus was on primary and secondary prevention programmes hence studies in which the respondents were community members of all age groups with no previously diagnosed MNS disorders, including those at risk (primary prevention) and with early stage of the illness (secondary prevention), were included.

Studies that involved patients that were admitted to or resident in a hospital or health facility setting were excluded because the review’s focus was on community based care provided outside of a health facility. There were no other restrictions, on the types of patients for whom data was extracted in the studies.

#### Types of participants -health care providers

Studies involving any lay health worker (paid or voluntary) including community health workers, village health workers, lay counsellors etc., were included. Lay community health workers in this review were defined as “any health worker working outside of a health care facility as part of a community based health care programme that met the following criteria: carried out functions related to health care delivery, were trained in some way in the context of the intervention and had no formal professional or paraprofessional certificated or tertiary education degree” [3]. Interventions in which a healthcare function was performed as an extension to a participant’s profession (for example teachers providing health promotion in schools) were excluded as were support groups (patient or peer) or family carers [3].

#### Types of interventions

Any community based health intervention for MNS disorders that utilised LHWs in the delivery of primary and secondary prevention programmes in LMICs was included. Interventions that involved tertiary prevention or that only targeted persons with fully established MNS disorders as measured by diagnostic instruments were excluded.

#### Types of studies

The literature review included studies that compared MNS prevention strategies delivered by LHWs with a control. Of these, only randomised controlled trials, controlled clinical trials, controlled prospective studies and, controlled before and after studies were considered for inclusion so that evidence from interventions provided plausibility or probability of effectiveness [15]. Uncontrolled before and after studies were excluded. Only studies undertaken in LMICs as defined by the World Bank Index were considered [6].

Interventions that involved “Head-to-head” comparisons of different LHW interventions and multi-faceted interventions that included LHWs and professionals working together but did not include a comparison group that allows for separate assessment of the effects of the LHW intervention, were excluded [3].

#### Types of outcome measures

Primary outcome: Studies were included if they assessed the proportion of the study population with improved mental health status, determined by changes in incidence or prevalence of MNS disorders measured by validated instruments.

Secondary outcomes: studies were included if they assessed any of the following outcome categories;

1) Consumer- oriented outcomes including: knowledge and understanding; health status and wellbeing; health behaviours such as changes in risk taking behaviour and other MNS treatment outcomes including adverse outcomes resulting from the intervention.

2) Health provider -oriented outcomes related to consultation processes such as rate of provision of services.

Outcomes were assessed depending on whether the intervention targeted primary or secondary prevention.The longest follow up period to the end of the intervention was the time point considered for the review. This list of outcomes was adapted from the list of outcomes of interest to the Cochrane Consumers and Communication Review Group [16].

#### Screening of studies

The potential relevance of all titles and abstracts identified from the databases was examined according to these inclusion and exclusion criteria by the first author. The screened titles and accompanying abstracts were imported into Endnote reference manager and duplicates removed. The titles and abstracts of citations were screened again to determine whether each paper met the predetermined criteria. In case of doubt, the full text of the article was retrieved and reviewed against the inclusion and exclusion criteria. The reference section of the articles was reviewed to identify any other potentially relevant studies.

### Data extraction and management

A data extraction form was developed using the data collection checklist developed by the Cochrane Effective Practice and Organisation of Care Review Group [17] and the Newcastle-Ottawa scale for assessment of bias in observational studies (18). Assessment of the risk that a randomised controlled or observational study over or under estimated the true intervention effect, was done using the Cochrane risk of bias tool (19) and the Newcastle-Ottawa scale [18] respectively. Using the Cochrane handbook recommendations, each criteria was judged ‘Yes’ indicating a low risk of bias, “No’ indicating high risk of bias, or ‘Unclear’ indicating either lack of information or uncertainty over the potential for bias [19]. In case of any missing information; efforts were made to contact the study authors.

### Data analysis

A qualitative synthesis of included studies was done with a focus on effectiveness of interventions and roles of LHWs. Studies that evaluated similar outcomes were grouped together and standardized mean differences and proportions were calculated. Effect sizes were then used to construct forest plots to illustrate individual study results, however because of clinical and methodological heterogeneity of included studies, there was no pooling of results and meta-analyses were not done [19].

## Results

The database searches yielded a total of 2104 records. Records from each database considered to be relevant to the review were identified on the basis of whether the study titles or abstracts reported on MNS disorders and whether studies had been conducted in LMICs. A total of 168 records were identified and 21 additional records obtained through searches of reference lists. Removal of duplicates resulted in 124 records, of which 45 were considered potentially relevant to the study question after further title and abstract screening.

**Figure 1:**
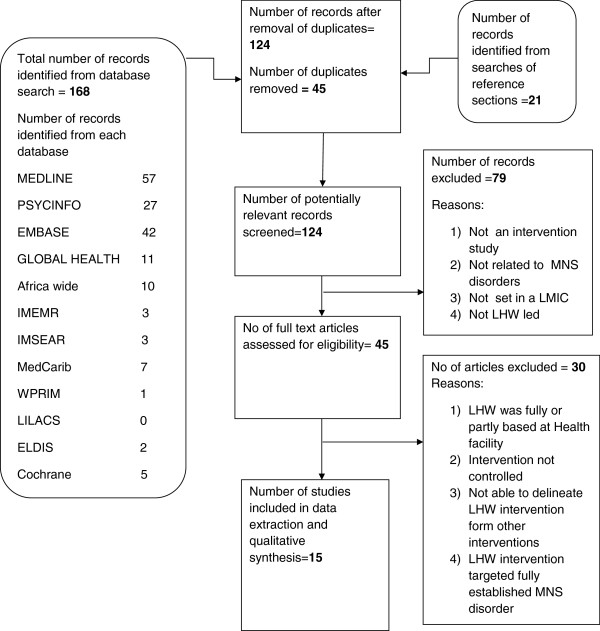
Flow chart showing the search and selection process.

The full text articles of these 45 records were retrieved and subjected to a more thorough screening against the inclusion criteria. A total of 20 articles reporting on 15 studies fulfilled the full inclusion criteria and were selected for the final analysis and data extraction. Figure 1 is a diagrammatic representation of the search and selection process as recommended by the preferred reporting items for systematic reviews and meta-analysis (PRISMA) statement [20].

### Study settings

The included studies represented six LMICs including India [21-23], Pakistan [24,25], Bangladesh [26], South Africa [27,28], Uganda [29] and Jamaica [30-34]. The largest number of studies (six) reported by 10 articles were conducted in Jamaica [30-39]. Six studies [21-24,26,29] were carried out in rural settings, with the majority carried out in urban settings (six of them in informal urban settlements) [25,27,28,30,31]. There were no studies identified that had been conducted in LMICs in South America, the Middle East or Europe (see Additional file 1: Table S1).

### Intervention characteristics

The majority of studies (93%, n = 14) involved a home visiting intervention [21,23-34]. Two studies involved an intervention that targeted participant groups gathered in a community area [22,26]. All interventions targeted specific population groups with none implemented at a general population level (Additional file 1: Table S1). Primary prevention and secondary prevention strategies were carried out in eleven [21-23,26-28,30-33] and four [24,25,29,34] studies respectively, considering the primary study outcome, Four studies employed both primary and secondary interventions with respect to the other targeted outcomes [21,23,27,28]. Studies targeting primary prevention of MNS disorders used selective [31] or indicated [21-23,26-28,30,32,33] preventive strategies. All six of the interventions that targeted child mental health outcomes involved primary prevention with four of the studies [26,30,32,33] using indicated prevention and only two studies [31] using selective primary prevention strategies. The activities carried out by LHWs varied in terms of time and type of engagement with study subjects. The pattern of activity ranged from home visits lasting half an hour [33,34] to home visits, group counselling and psychotherapy sessions lasting up to two hours [29]. Frequency of engagement with study participants varied from twice weekly [29] to once a month [21].

### Participants

The selection, training and supervision of LHWs varied between studies and information on some of these aspects was lacking for a number of the included interventions. In four of the studies, LHWs were recruited on the basis of being part of the community, literate, motivated to participate in the intervention and able to communicate with community members [22-24,28]. Most of the studies (67%, n = 10) used LHWs that did not have any previous training in health care. One study selected LHWs with previous mental health training [21] and a few studies (27%, n = 4) used LHWs with previous nutritional and health care training [30,31,34].

The description of the education level of LHWs varied from those with limited schooling, ability to read and write or completed primary education to some who had completed secondary school or university education [25,27-34]. This information is lacking for two of the studies [21,26] making it difficult to categorise the levels of education of LHWs with completeness. The duration of LHW training also varied, with the shortest training period being a week [21-23] and the longest lasting four months [28]. A majority of studies (80%, n = 12 ) reported on activities of supervision, monitoring or on-going support of LHWs by study team members e.g. through regular review meetings between LHWs and a supervisor, and these were as varied as the interventions carried out [22,23,25-33].

A wide range of intervention recipients were studied including infants [32,33] and the elderly [23]; twelve of the fifteen studies (81%) involved children and/or mothers, and pregnant women [25-28,30-34]. The sample sizes ranged from 58 individuals studied in a home visiting intervention [31] to 12431 individuals in a participatory intervention with women’s groups [22].

### LHW roles

In seven of the studies, LHWs provided psychosocial stimulation to children [26,30-34]. In one of these studies, LHWs provided nutritional supplements to children in addition to psychosocial stimulation [30]. Emotional and social support, psychotherapy and counselling were provided to study participants in another seven studies [21,23,24,27-29]. LHWs were involved in activities aimed at improving education and awareness in two of the studies; with one targeting women’s groups [22] and the other, which also involved provision of emotional support, targeting care givers [23].

### Outcomes

A minority of studies (13%, n = 2) did not have the evaluation of mental health status as the primary objective (27–28). Studies used a variety of tools to assess different mental health outcomes. The tools commonly used included the World Health Organisation wellbeing index (WHO-5), General Health Questionnaire (GHQ), Post Traumatic Stress Disorder (PTSD) Scales and the Zarit Burden scale. Interestingly, even among studies which had similar interventions such as those which involved psychosocial stimulation of children, the outcome measures varied (26, 30, 32–33). In the seven studies that assessed symptoms of depression, the Becks depressive inventory (21), the Aga Khan University Anxiety and Depression Scale (AKUADS) (24–25), the Structured Clinical Interview for Diagnostic and Statistical Manual of Mental Disorders (DSM-IV) diagnosis (SCID) (27–28), the Kessler 10 item questionnaire (22), the Edinburgh postnatal depression scale (28) and the Centre for Epidemiological Studies Depression scale (34) were all tools that were used.

Children’s mental development was assessed using a variety of tools dependent on the age of the child. Infants were assessed using the Griffiths mental development scale, intentional problem solving tests, behaviour rating scales and the Bayley scales of infant development (26, 30–33, 35–38). Tools used to assess older children and adolescents included the Wide Range Achievement Test (WRAT:26), Stanford Binet test, Wechsler intelligence scales for children (Revised), Peabody picture vocabulary test (PPVT), Ravens progressive matrices and the Wechsler Adult Intelligence Scales (WAIS) (30–31, 33, 35–39).

Other outcomes assessed included level of stimulation in the home, functional impairment, and disability and social support (23, 30, 33). Adverse outcomes were reported by only two studies and these related to number of suicide attempts (21) and mortality rates (23) of study participants.

Five studies reported on dichotomous outcomes (21–23, 27–28) with the rest of the studies reporting continuous outcome measures (mean scores and mean difference measures). An Additional file 2: Table S2 summarises the various outcome measures and corresponding measures of effect (see Additional file 2: Table S2).

### Measure of effectiveness

The studies included in the final analysis showed wide ranging heterogeneity with respect to study participants, setting, nature of intervention delivered, roles of LHWs and outcomes measured. For a number of studies p-values and/or confidence interval estimates were not provided, making judgement of study effectiveness difficult (Additional file 2: Table S2).

**Figure 2:**
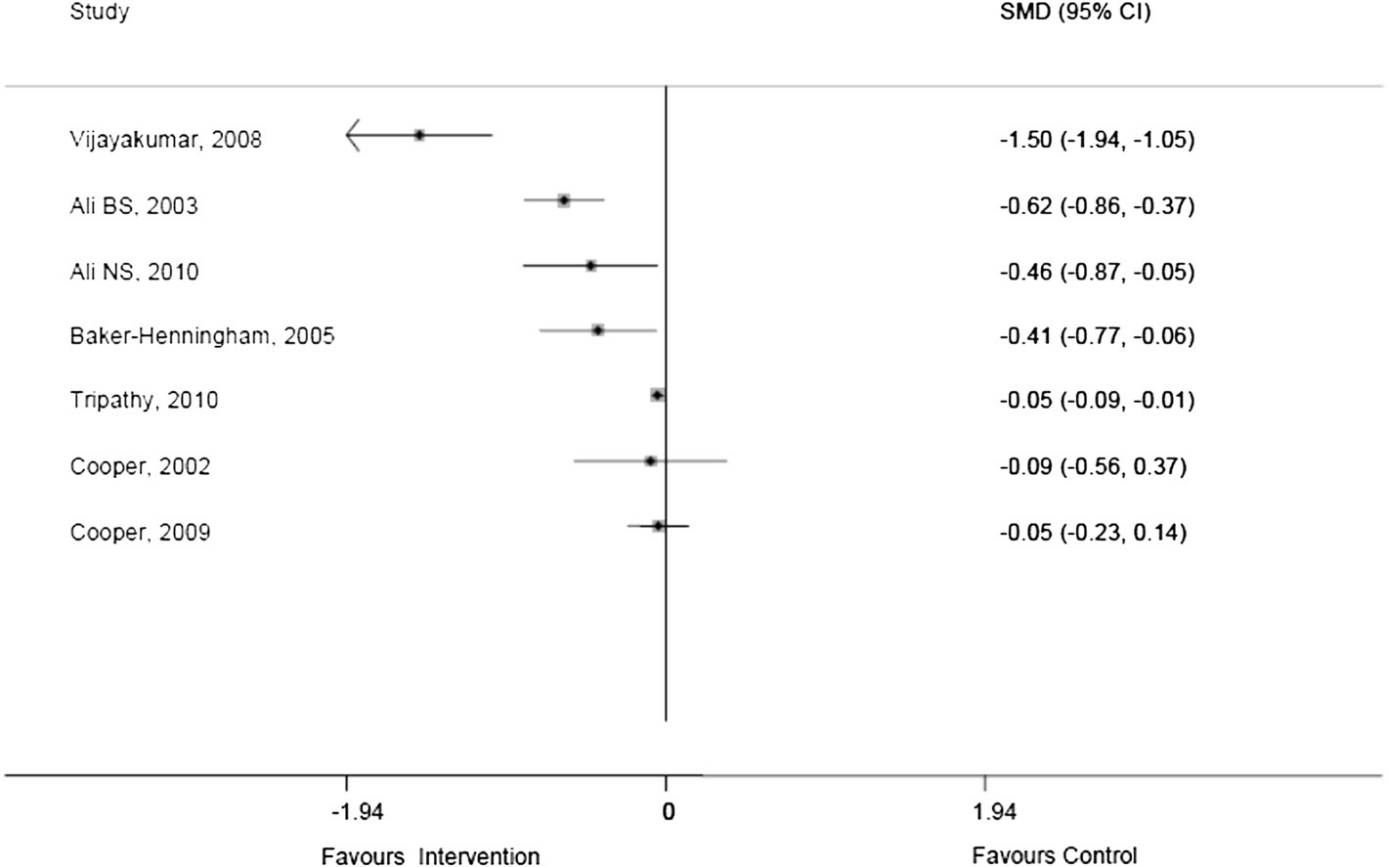
Forest plots of studies on depressive symptoms.

Though this heterogeneity precluded a meta-analysis, forest plots were constructed to provide a visual analysis of studies evaluating similar outcomes. Seven studies (47%) evaluated interventions aimed at reducing depressive symptoms in adults [21,22,24,25,27,28,34] with six of these targeting maternal depressive symptoms [22,24,25,27,28,34]. Two of these seven studies on adult depression showed statistically non-significant reductions (p > 0.05) in depressive symptoms [27,28] with the other five indicating statistically significant reductions (Figure 2).

**Figure 3:**
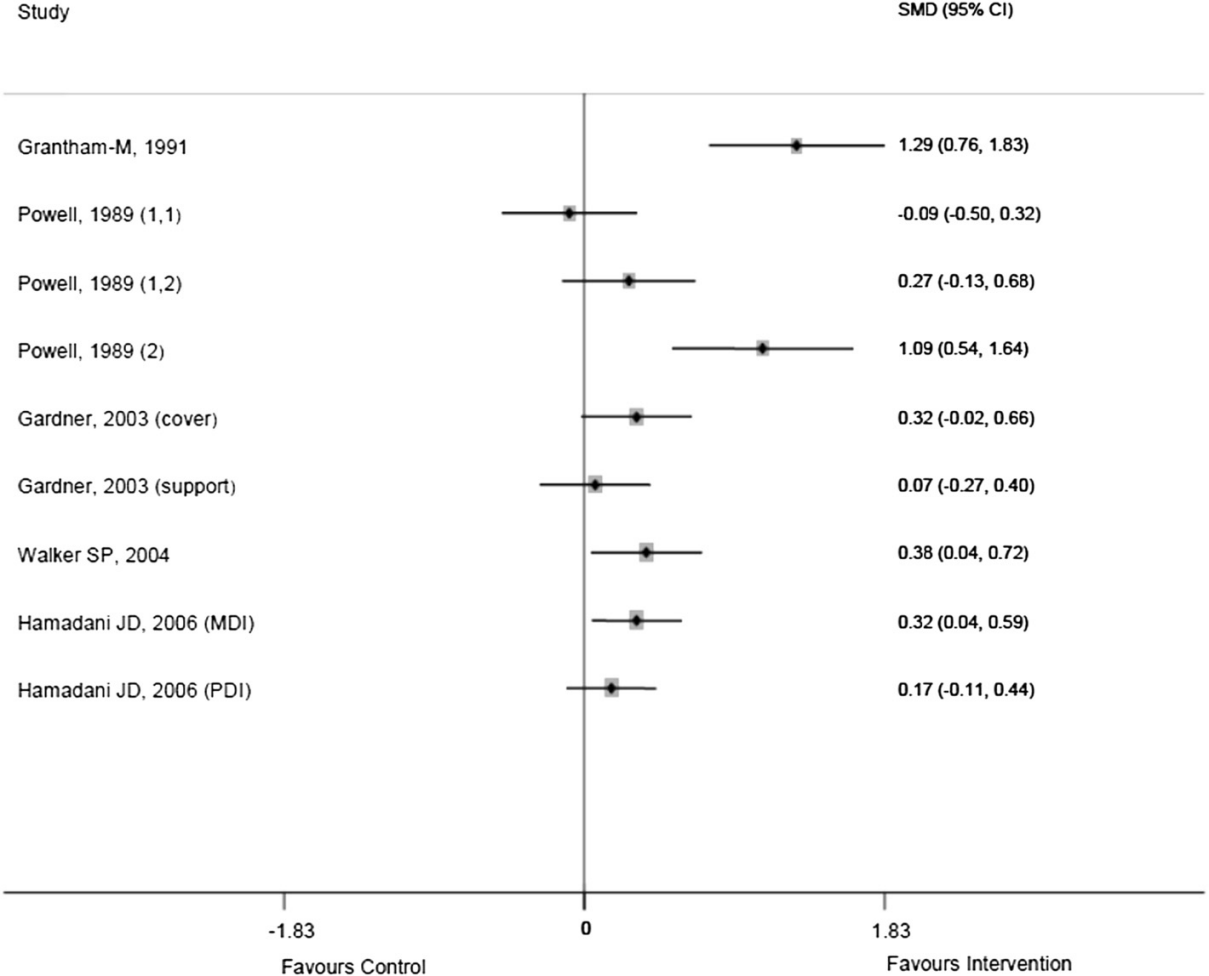
Forest plots of studies on child mental development.

Ten studies (62.5%) assessed aspects of child mental health including mental development, behaviour and mother child interaction [25-28,30-34]. Six of these studies evaluated changes in child mental development [26,30-34], however, information on child mental health outcomes was lacking in some of the studies [25,27,34]. Four of the studies evaluating child mental health indicated that the intervention was effective for most of the aspects assessed with two of them [30,33] showing effectiveness of interventions after long term follow up of children (Figure 3).

Two studies provided evidence of effectiveness of interventions that targeted symptoms of Post-Traumatic Stress Disorder (PTSD) [21,29]. The one study that assessed functioning of dementia patients and psychological burden of their caregivers indicated that the interventions were effective for both patient functioning and caregiver burden [23] (Additional file 2: Table S2).

### Methodological quality and risk of bias in included studies

Additional file 3: Table S3 presents the risk of bias assessment of included studies (see Additional file 3: Table S3). The key domains considered for risk of bias within a study were allocation concealment, blinding of outcome assessment and handling of incomplete outcome data [19]. Of the eleven randomised studies, seven were judged as having unclear risk of bias and four as having high risk of bias (Additional file 3: Table S3). Allocation concealment was unclear in eight and not done in three randomised studies. Blinding of outcome assessment was done in seven studies, unclear in three studies and not done in one study. It was unclear whether incomplete outcome data was handled by intention to treat analysis in five studies and clearly, not done in one study. Only one of the four non-randomised studies was classified as having unclear risk of bias [31] with the others having a high risk of bias either because of improper selection of the non-exposed cohort [21,27] or lack of a blinded assessment of outcome [21]. Appropriate statistical methods were used to control for confounding in all the non-randomised studies.

## Discussion

This review presents findings from 15 controlled interventions led by LHWs, of which 11 studies were randomised controlled trials. Studies were heterogeneous with respect to interventions and outcomes assessed, although there were outcomes common to some of the interventions including those related to child mental development and to symptoms of adult depression. Heterogeneity in the studies is also reflected in the settings, study participants, assessment tools, follow up periods and outcomes measured, and hence a meta-analysis was deemed inappropriate.

A variety of secondary outcomes related to the mental health of consumers were reported but were difficult to analyse across studies as they were specific to the interventions. Despite its importance, reporting on adverse outcomes such as mortality or symptom progression in study subjects was absent in all but two studies [21,23]. Most studies described the nature and pattern of the intervention, however there was insufficient reporting on health provider outcomes, particularly on fidelity of the interventions delivered. Knowing how the intervention was delivered is essential in determining how effective it could be if applied to another context [15,19].

### Evidence of effectiveness

Primary prevention includes selective, indicated and universal preventive strategies [8,9]. In this review, indicated preventive interventions were effective in reducing the burden of MNS disorders including depression and PTSD in three adult study populations [21-23]. Although no study used selective prevention of MNS disorders in adult populations, these findings are consistent with literature on studies conducted in developed countries showing that both selective and indicated interventions have been effective in reducing depressive symptoms in adults [9,10]. Four of the studies targeting child mental health outcomes, one using selective prevention [31] and three using indicated prevention [26,30,33], showed effectiveness of the interventions (Figure 3). Similar findings of effectiveness have been reported from primary prevention studies conducted in high income countries which targeted a range of developmental outcomes in infants and children [40-43].

Universal prevention entails the provision of an intervention at general population level and not on the basis of increased risk of a particular population [8-10]. Notably, none of the studies included in this review involved universal preventive strategies highlighting a research gap in LMICs, of LHW-led effectiveness studies that use this type of primary prevention. This could be in line with the observation that selective and indicated preventive interventions are more promising than universal preventive programmes which “often fail to reach those most in need, and expend their energy on those who do not need them” [44]. It is also worth noting that larger populations are likely to require more resources, hence universal programmes may not be the first choice for those designing preventive interventions in low resourced settings.

Even with results showing that primary prevention can be effective, emphasis is usually put on treatment of identified illnesses rather than primary prevention which is thought to be the only sustainable method for reducing the burden of mental disorders [9,10]. Importantly, one intervention which was effective in reducing depressive symptoms in women used a large sample size and could therefore provide evidence of effectiveness of large scale community (primary) prevention programs [22].

No study targeted secondary prevention of MNS disorders in children. Secondary prevention strategies mainly targeted adults with symptoms of MNS disorders and showed statistically significant reductions in prevalence [27,28] or mean scores of depressive symptoms [24,25,34] or PTSD symptoms [29]. It is possible that the review’s definition of secondary prevention, with a focus on treatment of early stage of illness and not a fully developed disorder, could have been restrictive and resulted in the exclusion of some studies from the review [8,12]. For example, studies by Carlo et al. (2013) which targeted children with birth asphyxia [45], Rahman et al. (2008) and Bolton et al. (2003) which targeted adults with major depressive disorder [46,47], were considered to be tertiary rather than secondary preventive strategies. Although the review’s inclusion criteria were strict in terms of study design, it is not clear that this would have restricted identification of literature on secondary prevention of MNS disorders in children using LHWs. Evidence from developed countries indicates effectiveness of secondary prevention of MNS disorders in children through school and home based interventions [10], and similar initiatives could be investigated in LMICs.

### Roles of lay health workers

Most studies (87.5%) involved a home visiting intervention though the mode of delivery varied across studies. Interventions were also different with respect to basic education, selection and training of LHWs which could have resulted in the diversity of LHW roles seen in the various studies. The level of education and or training determines the role(s) that LHWs have in a particular intervention [48]. This therefore makes it difficult to generalise their role in MNS disorder prevention programmes, however, based on the review’s findings, there is an indication that LHWs can provide psychosocial and psychological preventive interventions in LMICs.

In keeping with evidence from other LHW led interventions carried out in similar settings, the review findings indicate that MNS prevention interventions led by LHWs are effective for particular outcomes [48]. However, it is important to note that a majority of studies showing evidence of effectiveness used small sample sizes with only two studies [22,28] having large sample sizes, thereby raising questions about the delivery of these interventions to scale. In addition, all of the identified studies had either unclear or high risk of bias assessment bringing into question the internal validity of study findings and raising the need for cautious interpretation. On the other hand, although outcomes tended to be subjective, the internal validity of study results was improved through blinding of outcome assessment which reduces the risk of measurement bias.

A multitude of factors are thought to contribute towards effectiveness of an intervention led by LHWs. These include: health system factors such as supervision of LHWs; national political and socioeconomic factors; and community factors related to leadership, infrastructure and community empowerment [48]. Studies included in the review did not provide much information about these contextual factors. This makes it difficult to comment on the role they play in LHW delivery of MNS prevention interventions which would guide national ministries on the implementation of LHW led programmes.

The sustainability of community health worker programmes is a common challenge, probably as a result of the many factors which have a bearing on their successful implementation [48]. Six of the identified studies were conducted in the same setting over a long period of time and used a similar intervention with respect to the roles of LHWs involved (30–39). Though the generalizability of these results to other LMICs is limited, they reveal a consistency of effectiveness of psychosocial stimulation of children as an intervention over the long term. There was however, no evidence of effectiveness of nutritional supplements on child mental development in the long term, at least in Jamaica [35-38]. These particular interventions used LHWs who were part of the health care system and can provide information about the sustainability of interventions led by LHWs. A relationship with formal health services is known to be a key determinant of the effectiveness and sustainability of a community health worker programme [48], however, some of the interventions used LHWs who were not part of the health system or had not received any health care training [22-26,29]. Though study results indicated effectiveness of these interventions, questions remain about the long term future of similar programmes.

Much of the evidence in this review has been generated from randomised controlled trials that indicate a high probability of effectiveness, with less of the evidence provided by non-randomised studies which show plausibility of effectiveness of an intervention [15]. However, in assessing effectiveness of an intervention, it is important to consider not only what was achieved (effect sizes) but also how and why it was achieved as these aspects of the study may be important for generalizability of the results to other settings [49]. Though the quality of observational studies is considered to be lower than that of randomised studies, they can be valuable alternatives in situations where randomisation cannot be done like in some health care interventions at community level [10]. In LMICs therefore, evidence provided by well-designed non-randomised studies could be a useful indicator for the value of LHW led interventions and could also provide useful process data that is useful for improving programme implementation.

### Limitations

The review was subject to a number of limitations. Only literature published in the English language were considered for inclusion and these were largely from databases that contain peer reviewed published journal articles. The reviewer was not able to carry out extensive hand searches of unpublished literature and hence could have missed some relevant studies. To try and address the gaps in information in some studies, seven authors were contacted for additional information and one responded with the sufficient information.

Lack of a common terminology describing LHWs made the search and identification of relevant studies difficult, and could have resulted in some relevant information being missed, particularly in Latin America and Europe which were underrepresented in this review. However, every attempt was made to ensure that the search criteria were comprehensive enough to capture all potential MeSH terms and keywords related to LHWs.

All data extraction and synthesis was carried out by the first author alone thereby introducing the potential for bias. Only fifteen studies representing six countries were found for inclusion in the review hence limiting applicability of the results to other LMICs. The majority of studies had small sample sizes and were judged as having unclear or high risk of bias, which may bring into question the quality of evidence generated. The description of the target population in some of the studies was not adequate to allow definitive judgement on whether prevention was primary and/ or secondary. This could have resulted in some studies missing out that would otherwise have been included in the review. In such cases, authors were contacted for more information.

Most of the studies indicated a positive outcome for the intervention. However, sensitivity analyses to determine if statistical significance varied with different subgroup characteristics was not done. The reviewer would also have wished to investigate further for publication bias towards studies with positive findings. A number of included studies were conducted and reported by the same authors increasing the risk of citation bias. Nevertheless, a systematic methodology was followed in searching the literature and the review results are likely to be a true indication of the current available evidence for LHW led MNS disorder prevention interventions in LMICs.

## Conclusions

This review provides some evidence of effectiveness of using LHWs in MNS disorder prevention programmes. It also indicates that LHWs’ roles in such programmes could involve provision of psychological and psychosocial interventions. From a policy perspective, it would therefore seem that LHW led prevention programmes demonstrate potential for implementation in other LMICs.

However, only a few studies from a limited number of countries have been conducted and the evidence base is heterogeneous with respect to intervention design, target population and outcomes measured. In addition, most of the included studies evaluated small populations and were judged to have potential for bias, indicating that the evidence for effectiveness is weak.

A need therefore exists for similar and larger studies to be carried out in other settings in LMICs that also allow for recording of indicators related to adverse outcomes, delivery of care processes, fidelity of interventions and cost effectiveness. Such information is necessary for the comprehensive evaluation of the feasibility and impact of large scale mental, neurological and substance use disorder prevention programmes led by lay health workers in low and middle Income countries.

## Abbreviations

AKUADS: Aga Khan University anxiety and depression scale; BDI: Becks depressive inventory; CESD: Centre for epidemiological studies depression scale; CHW: Community health worker; DSM IV: Diagnostic and statistical manual of mental disorders fourth edition; ELDIS: Institute of development studies database; EMBASE: Excerpta medica database; EPOC: Effective practice and organisation of care; GHQ: General health questionnaire; HIV: Human immunodeficiency virus; HOME: Home observation for the measurement of the environment; IMEMR: Index medicus for Eastern Mediterranean database; IMSEAR: Index medicus for South East Asia region database; IQ: Intelligence quotient; LHW: Lay health worker; LILACS: Latin American and Caribbean literature on health sciences database; LMICs: Low and middle income countries; MC-HOME: Middle childhood home observation for the measurement of the environment; MDI: Mental development indices; MedCarib: Caribbean health sciences literature database; MEDLINE: International database for medical literature; MESH: Medical subject headings; MG: Monitoring group; MNS: Mental Neurological and substance use; NET: Narrative exposure therapy; OR: Odds ratio; PDI: Psychomotor development indices; PDS: Post traumatic stress disorder scale; PPVT: Peabody picture vocabulary test; PRISMA: Preferred reporting items for systematic reviews and meta-analysis; PsycINFO: Psychological information database 5; PTSD: Post traumatic stress disorder; SCID: Structured clinical interview for diagnostic manual of mental disorders; SDQ: Strengths and difficulties questionnaire; TC: Trauma counselling; VHW: Village health worker; WAIS: Wechsler’s adult intelligence scales; WHO: World health organisation; WHO-5: WHO wellbeing index; WPPSI-111: Wechsler preschool and primary scale of intelligence; WPRIM: Western Pacific region index medicus database; WRAT-26: Wide range achievement test.

## Competing interests

The authors declare that they have no competing of interest.

## Authors’ contribution

BM developed the initial draft and performed the database searches and screening for articles. NV, LS, DS participated in the drafting of the manuscript and helped with discussion of the review. SW provided statistical input. All authors read and approved the final manuscript.

## Pre-publication history

The pre-publication history for this paper can be accessed here:

http://www.biomedcentral.com/1472-6963/13/412/prepub

## Supplementary Material

Additional file 1: Table S1 Characteristics of included studies. Click here for file

Additional file 2: Table S2 Summary of findings from included studies. Click here for file

Additional file 3: Table S3 Risk of bias assessment. Click here for file
