# Supplementary material for: Roles and effectiveness of lay community health workers in the prevention of mental, neurological and substance use disorders in low and middle income countries: a systematic review
Source: BMC Health Serv Res. 2013 Oct 13;13:412. doi: 10.1186/1472-6963-13-412 (PMC3852794; doi:10.1186/1472-6963-13-412)
Supplement: Additional file 1: Table S1 — Characteristics of included studies. [file 1472-6963-13-412-S1.docx]

| Source | Setting | Study design & sample size | Inclusion criteria | Intervention | Targeted outcomes | Follow up |
| --- | --- | --- | --- | --- | --- | --- |
| (21)Vijayakumar, L. and M.S. Kumar, 2008. * | Homes in Rural India | Non Randomised Controlled Study  Intervention:45,  Control :57 | Non migrant adults of both sexes, who had lost at least one close family member. | Volunteers visited households monthly and provided emotional support to the bereaved family.  Control: Usual care | Levels of psychological distress, Symptoms of Depression and PTSD | 1 year |
| (22)Tripathy, P., et al., 2010. * | Community areas in Rural India. | Cluster Randomized controlled trial  Intervention :6452  Control :5979 | \| Women aged 15-49 years, who had given birth \| \| --- \| | Groups met monthly and took part in a participatory learning and action cycle facilitated by the CHW (Peer facilitator). Information about clean delivery practices and care-seeking behaviour was shared. Group members identified and prioritised maternal and new born health problems in the community, selected and implemented relevant strategies and assessed the results. Control: Usual care | Symptoms of Depression | 3 years |
| (23)Dias, A., et al., 2008. * | Homes in Rural India | Randomized controlled trial.  Intervention :41  Control :40 | Persons with Dementia and their caregivers | \| HCAs visited homes at least once a fortnight for six months, to improve the awareness and knowledge of family caregivers regarding dementia, to provide emotional support to caregivers, to maximise their care giving resources and to improve their care giving skill. Control: Usual care \| \| --- \| | Psychological distress | 6 months |
| (24)Ali, B.S., et al.,2003 | Homes in Rural Pakistan | Randomized Controlled Trial. Intervention :124  Control :150 | \| Women aged between 18 years and 50 years,. \| \| --- \| | Consenting women were assigned to trained counsellors. Supportive, cognitive and problem solving counselling was provided at client’s residence once a week. Control: Usual care | Symptoms of Anxiety and Depression | 8 weeks |
| \| **(25) Ali, N.S., et al.,2010** \| \| --- \| | Homes in informal urban settlements in Pakistan | Quasi Experimental Study  Intervention :62  Control :38 | Mothers of children aged 0-30 months | \| Women counsellors provided very basic cognitive behavioural therapy, supportive and problem-solving counselling. Sessions were conducted at the client's residence once a week. Control: Usual care \| \| --- \| | Symptoms of Anxiety and Depression | 8 weeks |
| (26) Hamadani, J.D., et al.2006. * | Homes in Rural Bangladesh | Randomized Controlled Trial  Intervention :104  Control :102 | Children with moderate and severe under nutrition aged 6-24 months | \| Weekly group meetings at the CNCs which included topics on child development and the importance of play. Individual sessions at respective homes, in which the play leaders demonstrated play activities to the mothers. Emphasised importance of praising, giving positive feedback and chatting with children. Control: Usual care \| \| --- \| | Child Mental development, Behaviour | 12 months |
| (27)Cooper, P.J., et al, 2002. * | Homes in informal urban settlements in South Africa | Non Randomised Controlled Study  Intervention :32  Control :32 | Women in third trimester of pregnancy | Home sessions to provide mother with emotional support and to encourage her in sensitive response interactions with her infant. Control: Usual care | Depressive symptoms, Maternal -Infant interactions | 6 months  post-partum |
| (28)Cooper, P. J., et al,2009. * | Homes in informal urban settlements in South Africa | Randomised Controlled Trial  Intervention :220  Control :229 | Women in third trimester of pregnancy | Home sessions to provide mother with emotional support and to encourage her in sensitive response interactions with her infant.  Control: Usual care | \| Depressive symptoms, mother Infant-  interactions and level of attachment. \| \| --- \| | 6 months and  18 months  post-partum |
| \| **(29) Neuner, F., et al., 2008.** \| \| --- \| | Homes in a refugee settlement in rural Uganda | Randomized Controlled Trial.  Intervention (NET):111  (TC):111  Control :55 | Adult refugees | \| Treatment types included Narrative Exposure therapy and Trauma counselling. Six treatment sessions, usually 2 per week.  Control: Usual care \| \| --- \| | Symptoms of Post-Traumatic Stress Disorder | 9 months |
| (30)Grantham McGregor, S.M. et al,1991. *   \| **(35)Grantham-Mc, S.M., et al., 1997, (36)Walker SP et al,2000, (37)Walker SP et al, 2005, (38) Walker, S.P., S. M. Chang, et al, 2006.** * \| \| --- \| | Homes in informal urban settlements in Jamaica | Randomized Controlled Study  Intervention :Stimulation=30,  Supplementation=32,  Both =32,Control :33  Follow up of a Randomized Controlled Study.  Intervention :122  Control :84 | Stunted children aged 9-24 months | \| Nutritional supplements delivered to the home each week by a CHW. Stimulated group took part in a programme of psychosocial stimulation; Mothers taught how to play with their children in such a way as to promote development.  Control: Usual care \| \| --- \| | Child Mental Development | 2 years  4 years,  7-8 years,  11-12 years,  14 years,  17-18 years |
| \| **(31)Powell, C. and S.Grantham-McGregor,1989*** \| \| --- \| | Homes in informal urban settlements in Jamaica | Controlled intervention study  Intervention :Monthly 45, Biweekly 49  Control :45 | Children with normal weight, aged between 6 and 30 months | \| CHWs visited homes once a week and taught mothers how to play with children in such a way as to promote development: Psychosocial stimulation. Control: Usual care \| \| --- \| | Child Mental Development | 2 years |
| \| **(31)Powell C & Grantham-McGregor .S, 1989.** * \| \| --- \| | Homes in informal urban settlements in Jamaica | Randomized Controlled Study  Intervention :29  Control :29 | Children with normal weight, aged between 6 and 30 months | CHWs visited homes once a week and taught mothers how to play with children in such a way as to promote development: Psychosocial stimulation. Control: Usual care | Child Mental Development | 1 year |
| (32) Gardner, J.M., et al.,2003.* | Homes in Urban Jamaica | Randomized Controlled Trial  Intervention :66  Control :69 | Low birth weight infants of gestational age from 36 to 37 weeks | \| CHWs visited the home once a week. Taught mothers how to increase the amount of ‘’conversation” between mother and infant, respond to their infant’s cues, show them affection, and focus their infant’s attention on the environment. Control: Usual care \| \| --- \| | Child Mental development, Cognition and behaviour | 7 months of age |
| (33)Walker, S.P., et al.2004. *  (39)Walker, S.P., et al.2010. * | \| Homes in Urban Jamaica \| \| --- \| | Randomized Controlled Trial  Intervention :66  Control :69  Controlled prospective study (Cohort).  Intervention :63  Control :67 | Low birth weight children born at term (≥37 weeks) | \| CHWs visited the home once a week. Taught mothers how to increase the amount of ‘’conversation” between mother and infant. During visit, CHW demonstrated play techniques to the mother and involved her in a play session with the child. Mothers were encouraged to use praise and positive reinforcement and discouraged from using physical punishment. Control: Usual care  From age 7 to 24 months visits lasted 30 minutes.  Control: Usual care \| \| --- \| | Child Mental development; cognition, intelligence and behaviour | 2 years  6 years |
| (34)Baker-Henningham, H., et al.,2005 | Homes in urban Kingston , St Andrew and St Catherine parishes, Jamaica | Cluster Randomised controlled trial.  Intervention :64  Control :61 | Mothers and their undernourished children aged between 9 months and 30 months | \| Weekly home visits by Community health aides, focused on improving child development by improving mothers’ knowledge and practices of child rearing and their parenting self-esteem. Demonstration of age appropriate play activities for the child. Control: Usual care \| \| --- \| | Symptoms of maternal depression | 1 year |

CHW: Community Health Worker. CNCs: Community Nutritional Centres. HCAs: Home Care Advisors. *Primary preventive interventions
