# Supplementary material for: Roles and effectiveness of lay community health workers in the prevention of mental, neurological and substance use disorders in low and middle income countries: a systematic review
Source: BMC Health Serv Res. 2013 Oct 13;13:412. doi: 10.1186/1472-6963-13-412 (PMC3852794; doi:10.1186/1472-6963-13-412)
Supplement: Additional file 2: Table S2 — Summary of findings from included studies. [file 1472-6963-13-412-S2.docx]

| Study | Outcomes measured (Tool used) | Control data | Intervention data | Measure of effect (95% CI) | P-value |
| --- | --- | --- | --- | --- | --- |
| (21)Vijayakumar., 2008 | Participants’ wellbeing (WHO-5) | 13.79±2.6 | 13.49±5.9 | Mean difference: -0.3(-2-1.4) | p=0.731 |
|  | Symptoms of Depression(BDI) | 21.88±3.5 | 13.11±7.9 | Mean difference: -8.7(-6.4-11.1) | p<0.001 |
|  | Psychological distress(GHQ) | 14.07±2.5 | 8.71±6.5 | Mean difference: -5.4(-3.5-7.2) | p<0.001 |
|  | Symptoms of Post-traumatic stress disorder(PTSD scale) | 10.51±2 | 6.16±3 | Mean difference: -4.4(-3.3-4.2) | p<0.001 |
|  | Number of suicide attempts | 3 | 0 | *No information provided* | *No information provided* |
| (22) Tripathy, P., N. 2010 | Proportion of women with moderate depression (Kessler 10 item questionnaire) | 10% | 5% | Adjusted OR:  0·43 (0·23–0·80) | *No information provided* |
|  | Proportion of women with severe depression(Kessler 10 item questionnaire) | <1% | <1% | Adjusted OR:  0·70 (0·15–3·31) | *No information provided* |
|  | Proportion of women with improved home care practices | *No information provided* | *No information provided* | *No information provided* | *No information provided* |
|  | Proportion of women with improved health care seeking behaviour | *No information provided* | *No information provided* | *No information provided* | *No information provided* |
| (23)Dias, A.,2008 | Proportion of caregivers with psychological distress (GHQ) | 77.5% | 77.5% | *No information provided* | *No information provided* |
|  | Mean caregiver psychological distress scores (GHQ) | 3.3(3.6) | 2.6(8.3) | Regression Coefficient:  -1.12 ( -2.07--0.17) | *No information provided* |
|  | Mean caregiver burden scores  (Zarit burden scale:ZBS) | 21.4(16.2) | 19(13) | Regression Coefficient:  -3.29 ( -6.78 0.20) | *No information provided* |
|  | Mortality of patients | 6 | 1 | *No information provided* | *No information provided* |
|  | Proportion of patients with normal functional abilities (Everyday Abilities scale for India: EAST) | 65% | 80.5% | *No information provided* | *No information provided* |
|  | Mean scores of functional abilities (EAST) | 8.7(2.2) | 8.5(2.3) | Regression Coefficient:  -0.36 (-2.83 -0.46) | *No information provided* |
| (24)Ali BS., 2003 | Participants’ mean scores of anxiety and depression symptoms(AKUADS) | 23.4(8.29) | 18.4(7.88) | *No information provided* | p<0.02 |
| (25)Ali, N. S.,2010 | Mean Anxiety and Depression symptom scores (AKUADS) | 14.1(8.8) | 11.0(5.2) | *No information provided* | P<0.001 |
| (26) Hamadani, J. D.,2006 | Infants Mean Development scores  (Bayley scales of Infant development: BSID-11) | MD1: 82.7 ± 17.2  PDI: 90.2 ± 17.4 | MD1: 87.6 ± 13.3  PDI: 93.0 ± 16.4 | Adjusted regression coefficients: 0.26  0.23 | p=0.007  p=0.06 |
|  | Infants Mean Behaviour ratings (Behaviour rating scale:  a) Emotional tone, b)Activity, c)cooperation d)vocalisation | *No information provided* | *No information provided* | *No information provided* | p=0.001,  b)p=0.005  c)p=0.03,  d)p=0.04 |
|  | Mothers knowledge of child rearing (Questionnaire) | *No information provided* | *No information provided* | *No information provided* | p<0.001 |
| (27)Cooper, P.J., 2002 | Prevalence of Maternal Depressive symptoms  (SCID) | 28% | 19% | *No information provided* | p=0.16 |
|  | Proportion of mothers showing infant interactions of at least moderate quality  (Video recordings) | *No information provided* | *No information provided* | *No information provided* | *No information provided* |
|  | Proportion of mothers who perceived the intervention as beneficial  (Adapted questionnaire) | *No information provided* | *No information provided* | *No information provided* | *No information provided* |
| (28)Cooper, P. J., 2009 | Proportion of mothers with Depressive symptoms  (SCID, Edinburgh post natal depression scale) | 15.5% | 10.9% | *No information provided* | p=0.21 |
|  | Proportion of infants with secure attachment(Video recordings) | 63% | 74% | OR: 1.70 | p=0.029 |
|  | Quality of mother- infant interactions; Sensitivity, Intrusiveness. (Video recordings, adapted questionnaire) | 5.31 (1.51)  8.17 (8.34) | 5.74 (1.88)  6.41 (7.27) | *No information provided* | p=0.043  p=0.023 |
| (29)Neuner,2008 | Score of Post-traumatic stress symptoms  (PDS) | 36.8% | 69.8% | *No information provided* | NET vs MG; p=0.017, TC vs MG; p=0.036 |
| (30)Grantham-McGregor, S.M.,1991 | Children’s Mean developmental scores (Griffiths Mental Development scale) | 92(8) | Stimulated: 103 (9) Supplemented: 100(13) Both: 107(12) | Regression coefficients:  Stimulated: 7.3(4.0-10.6),  Supplemented:  6.1(2.9-9.40) | p<0.05  *No information provided* |
|  | Mean Differences in mothers verbal IQ and index of stimulation(PPVT,HOME) | *No information provided* | *No information provided* | *No information provided* | *No information provide* |
| (35)Grantham-Mc,1997,(36) Walker SP et al,2000,(37) Walker SP ,2005,(38) Walker, S. P.,2006 | Children’s Mean developmental scores (WRAT:26,Stanford Binet Test, PPVT, Pegboard) | 73.2±8.2 | Stimulated: 76.9±9.2 Supplemented: 76.4±9.8, Both: 76.4±8.9 | *No information provided* | Stimulated vs control; p<0.05, Both vs control; p<0.01. |
|  | Mean Differences in mothers verbal IQ and index of stimulation(PPVT) | *No information provided* | *No information provided* | *No information provided* | *No information provided* |
|  | Children’s Mean IQ , Cognitive and Growth scores(Wechsler Intelligence Scales for children (Revised),  PPVT, Ravens Progressive matrices) | 65.4(11.8) | Stimulated: 71.7(13.6) Supplemented: 69.6(12.8)  Both: 71.5(13.8) | *No information provided* | p<0.05  p<0.05  p<0.01*,* |
|  | Mean Differences in mothers verbal IQ and index of stimulation(PPVT) | 83.1(19.5) | Stimulated: 83.8(21.5) Supplemented: 89.7(22.5)  Both: 89.3(23.7) | *No information provided* | *No information provided* |
|  | Mean IQ ,and Cognitive  Scores(WAIS,PPVT, Ravens Progressive matrices, WRAT) | -0.55(0.66) | Stimulated: 0.07(0.95)  Supplemented: -0.31(0.92)  Both:-0.09(1.00) | Regression Coefficient:  0.38(0.06-0.71) | p=0.02. |
|  | Mean scores of Psychosocial outcomes ;A: Anxiety, D:Depression, S:Self-esteem, N:Antisocial behaviour, T:Attention deficit, C:Cognitive problems, H:hyperactivity, O:Oppositional behaviour.(Questionnaires) | A:15.8,D:7.3,S:25.2,N:5.8,  T:15.0,C:7.1,H:4.6,O:7.4 | Supplemented; A:16.9,D:23.2,S:13.6,N:6.4,T:4.8,  C:7.9  Stimulated; A:13.5,D:4.4,S:26.3,N:4.4,T:9.6,  C:5.9,H:4.8,O:6.0  Both; A:13.7,D:25.1,S:4.4,N:12.0,T:5.4,  C:4.3,H: 6.0.O:*No information provided* | Regression Coefficient:  A:-2.81(-5.02 to -0.61),  D:-0.43(-0.78 to -0.07),  S:1.55 (0.08 to 3.02),  T:-3.34(-6.48 to -0.19),  C,H,O: No information provided | A: p=0.01,  D: p=0.02  S: p=0.04  T: p=0.04,  C,H,O: *No information provided* |
| (31)Powell C,1989 | Children’s Mean developmental scores  (Griffiths Mental Development scale) | 100.3±9.4 | Monthly: 99.4±10.3  Bi-weekly: 102.9±9.7 | *No information provided* | biweekly vs control: p<0.01, biweekly vs monthly: p<0.02 |
| (31)Powell C,1989 | Children’s Mean developmental scores  (Griffiths Mental Development scale) | 98.6±9.6 | 109.6±10.6 | *No information provided* | p<0.02 |
| (32)Gardner, J. M., 2003. | Infants mean cognition and behaviour scores(Intentional problem solving ability tests, behaviour rating scales) | Cover: 1.9 (1.4-2.3)  Support: 1.6 (1,2-2.0) | Cover: 2.5 (2.0-3.0)  Support: 1.7 (1.3-2.1) | *No information provided* | P<0.05,  Less happy: p<0.05, less cooperative: p<0.01 |
| (33)Walker, S.P., 2004. | Infants mean development scores  (Griffiths Mental Development scale) | 108.5±7.2 | 111.0±5.9 | *No information provided* | *No information provided* |
| (39)Walker, S. P., 2010 | The level of stimulation in the home (HOME) | 32.2±7.2 | 34.9±7.7 | *No information provided* | p<0.05 |
|  | Mean cognition scores (WPPSI-111, ,PPVT) | 78.7 (75.9–81.6)  45.4 (41.2–49.6) | 81.0 (78.5–83.5)  43.5 (39.7–47.3) | Regression coefficient:  2.28(-0.58-5.46) | p=0.2 |
|  | Mean behaviour scores (Strengths and difficulties questionnaire: SDQ) | 16.8 (15.2–18.3) | 14.5 (13.1–16.0) | Regression coefficient:  -1.93(-7.38-3.55) | p=0.5 |
|  | Mean stimulation in the home scores  (MCHOME) | *No information provided* | *No information provided* | Regression coefficient:  -2.21(-4.13 to -0.10) | p=0.029 |
| (34) Baker-Henningham, H. 2005. | Mean Maternal Depression scores (Centre for Epidemiological studies Depression scale; CESD) | 5.3 (2.1) | 4.3 (2.7) | Regression coefficient:  -0.98 (-1.53 to -0.41). | *No information provided* |

AKUADS, Aga Khan University Depression and Anxiety Scale. BDI,Becks depressive inventory.GHQ, General health questionnaire.HOME, Betty Caldwell index of stimulation in the home. IQ, Intelligent Quotient. MCHOME, Middle Childhood Home Observation for the Measurement of the Environment. MDI, Mental development indices.MG, Monitoring group. NET, Narrative Exposure Therapy. OR, Odds Ratio .PDI,Psychomotor development indices. PDS, Post traumatic stress disorder scale.PPVT, Peabody Picture Vocabulary test.TC, Trauma Counselling. WAIS, Wechsler Adult Intelligence Scales. WHO-5, WHO wellbeing index. WPPSI-111,Wechsler Preschool and Primary Scale of Intelligence. WRAT 26, Wide Range Achievement Test.
