# Supplementary material for: Roles and effectiveness of lay community health workers in the prevention of mental, neurological and substance use disorders in low and middle income countries: a systematic review
Source: BMC Health Serv Res. 2013 Oct 13;13:412. doi: 10.1186/1472-6963-13-412 (PMC3852794; doi:10.1186/1472-6963-13-412)
Supplement: Additional file 3: Table S3 — Risk of bias assessment. [file 1472-6963-13-412-S3.docx]

Table 3: Risk of Bias Assessment

| Domain | Selection Bias | | Performance Bias | | Detection Bias | Attrition Bias | Reporting Bias | Other Sources of Bias | Summary assessment of Risk of Bias |
| --- | --- | --- | --- | --- | --- | --- | --- | --- | --- |
| Study (Randomised studies) | *Was Random Sequence Generation done?* | *Was Allocation concealment done?* | *Was Blinding of Subjects done?* | *Was Blinding of Personnel (Research) done?* | *Was blinding of Outcome assessment done?* | *Was Incomplete outcome data present and was it handled?(including whether intention to treat( ITT) analysis was done)* | *Was Selective outcome Reporting examined for?* | Whether measurement  of baseline characteristics  was done? |  |
| (22) Tripathy, 2010 | Yes | No | No | No | No | Yes | Unclear | Yes | High Risk of Bias |
| (23) Dias, 2008 | Yes | Unclear | No | Unclear | Unclear | Unclear | Unclear | Yes | Unclear risk of bias |
| (24)Ali 2003 | Unclear | Unclear | No | Yes | Yes | Unclear | Unclear | Yes | Unclear risk of bias |
| (26)Hamadani,  2006 | Yes | Unclear | No | Unclear | Yes | Yes | Unclear | Yes | Unclear risk of bias |
| (28) Cooper 2009 | Yes | Unclear | Yes | Yes | Unclear | Unclear | Unclear | Yes | Unclear risk of Bias |
| (29)Nuener,  2008 | Unclear | No | No | Unclear | Unclear | Unclear | Yes | Yes | High Risk of Bias |
| (30)Grantham M,1991 | Yes | Unclear | Unclear | Yes | Yes | No | Unclear | Yes | High risk of Bias |
| (31)Powell,  1989, study 2 | Yes | Unclear | Unclear | Unclear | Yes | Yes | Unclear | Yes | Unclear risk of bias |
| (32) Gardner 2003 | Yes | Unclear | Unclear | Yes | Yes | Yes | Unclear | Yes | Unclear risk of bias |
| (33)Walker 2004 | Yes | Unclear | Unclear | Yes | Yes | Unclear | Unclear | Yes | Unclear risk of bias |
| (34)Baker-Henningham, H., (2005). | Yes | No | No | Yes | Yes | No | No | Yes | High Risk of Bias |

| Domain | Selection Bias | | | | | | Bias related to Outcomes | | | Confounding | | Summary assessment of  Risk of Bias | |
| --- | --- | --- | --- | --- | --- | --- | --- | --- | --- | --- | --- | --- | --- |
| Study (Non Randomised studies) | Representativeness of the exposed cohort | Proper selection of the non-exposed cohort | Ascertainment of exposure | Demonstration that outcome of interest was not present at start of study | Comparability of cohorts on the basis of design or analysis | Assessment of outcome(independent blind assessment , record linkage, self report) | | Was follow-up adequate and long enough for outcomes to occur*?* | Were appropriate statistical methods used to control for confounding factors? | |  | |  |
| (21) Vijayakumar L, Kumar M.S 2008 | Unclear | No | Yes | Unclear | Unclear | No | | Yes | Yes | | High risk of Bias | |  |
| (25)Ali, N. S.,et al. ,2010 | Unclear | Unclear | Yes | Unclear | No | No | | No | Yes | | High risk of Bias | |  |
| (27)Cooper, P.J., et al., 2002, | No | No | Yes | Unclear | No | Yes | | Yes | Yes | | High risk of Bias | |  |
| (31)Powell,1989, study 1 | Unclear | Unclear | Yes | Yes | Unclear | Yes | | Unclear | Yes | | Unclear risk of Bias | |  |
